# SARS-CoV-2 omicron BA.5 and XBB variants have increased neurotropic potential over BA.1 in K18-hACE2 mice and human brain organoids

**DOI:** 10.3389/fmicb.2023.1320856

**Published:** 2023-11-23

**Authors:** Romal Stewart, Kexin Yan, Sevannah A. Ellis, Cameron R. Bishop, Troy Dumenil, Bing Tang, Wilson Nguyen, Thibaut Larcher, Rhys Parry, Julian De Jun Sng, Alexander A. Khromykh, Robert K. P. Sullivan, Mary Lor, Frédéric A. Meunier, Daniel J. Rawle, Andreas Suhrbier

**Affiliations:** ^1^QIMR Berghofer Medical Research Institute, Brisbane, QLD, Australia; ^2^Clem Jones Centre for Ageing Dementia Research, Queensland Brain Institute, University of Queensland, Brisbane, QLD, Australia; ^3^INRAE, Oniris, PAnTher, APEX, Nantes, France; ^4^School of Chemistry and Molecular Biosciences, University of Queensland, St Lucia, QLD, Australia; ^5^GVN Centre of Excellence, Australian Infectious Disease Research Centre, Brisbane, QLD, Australia

**Keywords:** SARS-CoV-2, omicron, XBB, BA.5, K18-hACE2, brain, organoid

## Abstract

The reduced pathogenicity of the omicron BA.1 sub-lineage compared to earlier variants is well described, although whether such attenuation is retained for later variants like BA.5 and XBB remains controversial. We show that BA.5 and XBB isolates were significantly more pathogenic in K18-hACE2 mice than a BA.1 isolate, showing increased neurotropic potential, resulting in fulminant brain infection and mortality, similar to that seen for original ancestral isolates. BA.5 also infected human cortical brain organoids to a greater extent than the BA.1 and original ancestral isolates. In the brains of mice, neurons were the main target of infection, and in human organoids neuronal progenitor cells and immature neurons were infected. The results herein suggest that evolving omicron variants may have increasing neurotropic potential.

## Introduction

The SARS-CoV-2 omicron lineage diverged considerably from earlier variants of concern, with the evolutionary origins remaining unclear, with the closest genetic common ancestor dating back to mid-2020 (Du et al., 2022; Mallapaty, 2022). Omicron viruses have spread faster globally than any previous variants. The BA.5 sub-lineage became the dominant SARS-CoV-2 virus in many countries (Tanne, 2022), with XBB now spreading rapidly (Yue et al., 2023). Long-COVID is now well described for many variants of concern (Davis et al., 2023), and also occurs after infection with BA.5 (Qasmieh et al., 2023). Neurological and psychiatric manifestations represent a major component of COVID-19 and long-COVID (Islam et al., 2022; Monje and Iwasaki, 2022; Xu et al., 2022; Davis et al., 2023; Proal et al., 2023; Shuai et al., 2023) and these remain for patients infected with omicron viruses (Chen et al., 2022; Cloete et al., 2022; Ludvigsson, 2022; Taquet et al., 2022), although extensive data specifically for BA.5 or XBB neurological manifestations have not yet emerged. The large number of changes in spike for omicron and omicron sub-lineages has rendered vaccination (Branche et al., 2022; Surie et al., 2022; Hansen et al., 2023) many monoclonal antibody treatments (Takashita et al., 2022), and prior exposure with other variants (Suryawanshi et al., 2022) less protective.

There is increasing evidence for brain abnormalities in COVID-19 patients (Douaud et al., 2022; Graham et al., 2022; Karpiel et al., 2022; Ledford, 2022; Pelizzari et al., 2022; Sanabria-Diaz et al., 2022). Encephalitis is well documented, usually in hospitalized COVID-19 patients with severe disease, with encephalitis predisposing to poor outcomes and a higher risk of mortality (Siow et al., 2021; Altmayer et al., 2022; Chakraborty and Basu, 2022; Islam et al., 2022; Ong et al., 2023). The mechanism(s) whereby brain pathology/immunopathology and/or neuropathology might manifest in COVID-19 patients remains to be fully characterized, with the systemic cytokine storm likely involved, but direct brain infection also implicated in some COVID-19 and long-COVID patients (Zhang et al., 2020; Burks et al., 2021; Andrews et al., 2022; Aschman et al., 2022; Douaud et al., 2022; Fernandez-Castaneda et al., 2022; Rutkai et al., 2022; Samudyata Oliveira et al., 2022; Bauer et al., 2022a; Proal et al., 2023; Shuai et al., 2023; Silva et al., 2023). A range of studies have now shown brain infection in COVID-19 patients (see Supplementary Table S1 for a full list and summary of findings). For instance, viral RNA or protein was detected in the brains of 20–38% of patients that died of COVID-19 (Matschke et al., 2020; Serrano et al., 2022). A number of groups have also reported detection of viral RNA in cerebrospinal fluid of COVID-19 patients (Supplementary Table S1), including patients infected with omicron virus strains (Dang et al., 2022). Finally, COVID-19-associated damage to the brain is also likely to be associated with neurological manifestations of long-COVID (Davis et al., 2023; De Paula et al., 2023; Ferrucci et al., 2023; Rothstein, 2023), and such damage may also contribute to the continuing excess deaths arising from the COVID-19 pandemic (De Hert et al., 2021; Li et al., 2021; Msemburi et al., 2023).

Controversy surrounds the issue of whether BA.5 has altered pathogenicity compared with earlier omicron variants. Some hamster and K18-hACE2 mouse studies have suggested increased lung infection/pathology for BA.5 (Kimura et al., 2022; Imbiakha et al., 2023; Ong et al., 2023), whereas other studies using these models argued that the reduced pathogenicity seen for the early omicron variants (Shuai et al., 2022) was retained by BA.5 (Rizvi et al., 2022; Uraki et al., 2022) and XBB (Tamura et al., 2023). Similarly, while some human studies report increased pathogenicity for BA.5 (Kouamen et al., 2022; Hansen et al., 2023; Kang et al., 2023; Russell et al., 2023), others report no significant changes (Wolter et al., 2022; Davies et al., 2023). XBB variants appear to have increased transmission potential (Islam et al., 2023), as well as enhanced receptor binding affinity, although the implications for pathogenicity remain to be established (Yue et al., 2023). Assessments of pathogenicity of new variants in human populations are complicated by the overall rising levels of protective immunity due to vaccinations and/or past infections, which would tend to reduce the clinical severity for later COVID-19 waves (Sigal, 2022; Wolter et al., 2022). Perhaps also pertinent for any such assessments is the diversifying pattern of COVID-19 and long-COVID disease manifestations (Kouamen et al., 2022; Proal et al., 2023; Shuai et al., 2023), with involvement of non-pulmonary organs/systems increasingly being recognized (Nchioua et al., 2022; Davis et al., 2023; El-Kassas et al., 2023; Normandin et al., 2023; Proal et al., 2023; Shuai et al., 2023).

The K18-hACE2 mouse model represents a model of severe COVID-19 that significantly recapitulates the lung pathology and inflammatory pathways seen in humans, and has been widely used for assessing new interventions and for studying SARS-CoV-2 biology (Yinda et al., 2021; Bishop et al., 2022; Dong et al., 2022; Da Silva Santos et al., 2023). Infection of K18-hACE2 mice with original ancestral isolates via intranasal inoculation, usually results in fulminant and lethal brain infections, with virus likely entering the brain via the olfactory epithelium, across the cribriform plate and into the olfactory bulb (Carossino et al., 2022; Dumenil et al., 2022; Olivarria Gema et al., 2022; Rothan et al., 2022; Vidal et al., 2022; Morgan et al., 2023). This route of entry into the brain has been implicated in a non-human primate study (Jiao et al., 2021), hamsters (De Melo et al., 2023) and may also be relevant for COVID-19 patients, although the fulminant brain infection seen in K18-hACE2 mice is not a feature of COVID-19 in humans (Bulfamante et al., 2020; Matschke et al., 2020; Awogbindin et al., 2021; Meinhardt et al., 2021; Beckman et al., 2022; Serrano et al., 2022). Neurons represent a target of SARS-CoV-2 infection in brains of K18-hACE2 mice (Rothan et al., 2022; Seehusen et al., 2022; Morgan et al., 2023), non-human primates (Beckman et al., 2022), and hamsters (De Melo et al., 2023), with infection of neurons also observed in COVID-19 patients (Song et al., 2021; Shen et al., 2022; Emmi et al., 2023). Reduced brain infection and the ensuing reduction in mortality after infection of K18-hACE2 mice with omicron BA.1 (Halfmann et al., 2022; Shuai et al., 2022; Tarres-Freixas et al., 2022) has been viewed as evidence that these viruses are less pathogenic (Shrestha et al., 2022; Sigal, 2022).

Herein we characterize BA.5 and XBB infection in model systems that allow robust investigation of neurotropic potential. We illustrate and characterize the increased levels of brain infection, pathology and mortality for BA.5 and XBB versus BA.1 infected K18-hACE2 mice. Infection of brain organoid systems with SARS-CoV-2 is well described (Song et al., 2021; Hou et al., 2022; Mesci et al., 2022), and we show herein that BA.5 productively infected human cortical brain organoids significantly better than BA.1. These results indicate that BA.5 and XBB may have increased neurotropic potential over BA.1.

## Methods

### Ethics statements and regulatory compliance

All mouse work was conducted in accordance with the Australian code for the care and use of animals for scientific purposes (National Health and Medical Research Council, Australia). Mouse work was approved by the QIMR Berghofer MRI Animal Ethics Committee (P3600). All infectious SARS-CoV-2 work was conducted in the BioSafety Level 3 (PC3) facility at the QIMR Berghofer MRI (Department of Agriculture, Fisheries and Forestry, certification Q2326 and Office of the Gene Technology Regulator certification 3,445). Breeding and use of GM mice was approved under a Notifiable Low Risk Dealing (NLRD) Identifier: NLRD_Suhrbier_Oct2020: NLRD 1.1(a). Mice were euthanized using carbon dioxide.

Collection of nasal swabs from consented COVID-19 patients was approved by the QIMR Berghofer Medical Research Institute Human Research Ethics Committee (P3600) and by the University of Queensland HREC (2022/HE001492).

### SARS-CoV-2 isolates

An original ancestral (Wuhan) strain isolate, SARS-CoV-2_QLD02_ (hCoV-19/Australia/QLD02/2020) (GISAID accession EPI_ISL_407896) was kindly provided by Dr. Alyssa Pyke and Fredrick Moore (Queensland Health Forensic & Scientific Services, Queensland Department of Health, Brisbane, Australia; Rawle et al., 2021). BA.1 and BA.5 omicron isolates were obtained at QIMR Berghofer MRI from nasal swabs from consented COVID-19 patients (Yan et al., 2022; Morgan et al., 2023) that were seeded onto Vero E6 cells (ATCC C1008). Infected Vero E6 cells analyzed by RNA-Seq and viral genomes *de novo* assembled using Trinity v 2.8.4. The omicron BA.1 isolate, SARS-CoV-2_QIMR01_ (SARS-CoV-2/human/AUS/QIMR01/2022), belongs to the BA.1.17 lineage (GenBank: ON819429 and GISAID EPI_ISL_13414183; Yan et al., 2022; Morgan et al., 2023). The omicron BA.5 isolate, SARS-CoV-2_QIMR03_ (SARS-CoV-2/human/AUS/QIMR03/2022) belongs to the BE.1 sublineage (GenBank: OP604184.1). The XBB isolate (SARS-CoV-2_UQ01_) was obtained from nasopharyngeal aspirates of consented COVID-19 patient at the University of Queensland using Vero E6-TMPRSS2 cells (Amarilla et al., 2021). The isolate (deposited as hCoV-19/Australia/UQ01/2023; GISAID EPI_ISL_17784860) is XBB.1.9.2.1.4 (Pango EG.1.4) a recombinant of BA.2.10.1 and BA.2.75.

Virus stocks were propagated in Vero E6 cells, viral stocks and tissue culture supernatants were checked for endotoxin (Johnson et al., 2005) and mycoplasma (MycoAlert, Lonza; La Linn et al., 1995). Viruses were tittered using CCID_50_ assays (Yan et al., 2021).

### Mouse infection and monitoring

K18-hACE2 mice (strain B6.Cg-Tg(K18-ACE2)2Prlmn/J, JAX Stock No: 034860) were purchased from The Jackson Laboratory (2023), United States, and were maintained in-house as heterozygotes by backcrossing to C57BL/6 J mice (Animal Resources Center, Canning Vale WA, Australia) as described (Bishop et al., 2022). Heterozygotes were inter-crossed to generate a homozygous K18-hACE2 transgenic mouse line. Genotyping was undertaken by PCR and sequencing across a SNP that associates with the hACE2 transgene to distinguish heterozygotes [TTTG(A/C)AAAC] from homozygotes (TTTGCAAAC). The mice were held under standard animal house conditions [for details see (Yan et al., 2022)] and female mice (≈10–20 weeks of age) received intrapulmonary infections delivered via the intranasal route with 5 × 10^4^ CCID_50_ of virus in 50 μL RPMI 1640 while under light anesthesia as described (Dumenil et al., 2022). Each group of mice within an experiment had a similar age range and distribution, with the mean age for each group not differing by more than 1 week. Mice were weighed and overt disease symptoms scored as described (Dumenil et al., 2022). Mice were euthanized using CO_2_, and tissue titers determined using CCID_50_ assays and Vero E6 cells as described (Rawle et al., 2021; Dumenil et al., 2022).

### Maintenance and expansion of human induced pluripotent stem cells

The human-induced pluripotent cells (hiPSCs) used in this study were reprogrammed from adult dermal fibroblasts (HDFa, Gibco, C0135C) using the CytoTune-iPS 2.0 Sendai Reprogramming Kit (Invitrogen, A16518; Oikari et al., 2020). They were cultured on human recombinant vitronectin (Thermo Fisher Scientific) coated plates in StemFlex medium (Thermo Fisher Scientific) according to the manufacturer’s guidelines.

### Generation of human cortical organoids

On day 0 of organoid culture, hiPSCs (less than passage 50) were dissociated with StemPro Accutase (Thermo Fisher Scientific) to generate a cell suspension. Cells were plated 5,000/well into an ultra-low-binding 96-well plate (Corning) in StemFlex media supplemented with 10 μM ROCK inhibitor Y-27632 (STEMCELL Technologies, Vancouver, Canada). From days 1–5, media was changed daily with StemFlex medium supplemented with 2 μM Dorsomorphine (Abcam) and 10 μM SB-431542 (Stemcell technologies). On day 5, the medium was replaced with a Neuro-induction medium consisting of DMEM/F12 (Thermo Fisher Scientific), 1% N2 Supplement (Thermo Fisher Scientific), 10 μg/mL heparin (STEMCELL Technologies), 1% penicillin/streptomycin (Thermo Fisher Scientific), 1% Non-essential Amino Acids (Thermo Fisher Scientific), 1% glutamax (Thermo Fisher Scientific) and 10 ng/mL FGF2 (Stemcell Technologies). On day 7, organoids were embedded in Matrigel (Corning), transferred to an ultra-low-binding 24-well plate (Corning) (one organoid per well), and continued to grow in Neuro-induction medium for three more days. On day 10, organoids were supplemented with differentiation medium, consisting of Neurobasal medium, 1% N2, 2% B27 supplements (Thermo Fisher Scientific), 0.5% Penicillin/Streptomycin, 1% Glutamax, 1% Non-essential Amino Acids, 50 μM of 2-mercaptoethanol (Merck), 2.5 μg/mL Insulin (Merck), 1% Knockout Serum Replacement (Thermo Fisher Scientific), 10 ng/mL FGF2, 1 μM CHIR99021 (Stemcell Technologies) and placed in a CelVivo Clinostar incubator (Invitro Technologies) (24 organoids per Clinoreactor) spinning at 20 rpm. All media changes from 10 days onwards were performed every other day.

### Infection of cortical brain organoids

Organoids (30 days old) were transferred from each Clinoreactor into an ultra-low-binding 24-well plate (one organoid per well), infected with various SARS-CoV-2 strains at a multiplicity of infection (MOI) of 1 and placed within a humidified tissue culture incubator at 37C, 5% CO_2_ for 2 h. Organoids were then washed twice with media, transferred into 50 mm LUMOX gas exchange dishes (SARSTEDT) (4 organoids per dish) containing 7 mL of differentiation media, and placed within a humidified tissue culture incubator at 37°C, 5% CO_2_ for 4 days. Supernatants were collected at the indicated days and titers measured by CCID_50_ assays as described above. At 4 dpi organoids were harvested and formalin fixed for histology and immunohistochemistry, or processed for RNA-Seq.

### Histology and immunohistochemistry

H&E staining of formalin fixed paraffin wax embedded tissue sections was undertaken as described (Amarilla et al., 2021; Rawle et al., 2021). Immunohistochemistry was undertaken as described using the in-house developed anti-spike monoclonal antibody, SCV-1E8 (Morgan et al., 2023).

### Dual labeling fluorescence immunohistochemistry

Paraffin embedded K18-hACE2 mouse brains were sectioned on a rotary microtome into 10 μm sagittal sections. Sections were dewaxed and antigen retrieval was performed in Antigen Recovery Solution (citrate buffer solution consisting of 10 mM sodium citrate, 0.05% SDS 0.01%, pH 6.0) at 90°C for 10 min in a BioCare decloaker. Slides were blocked with blocking buffer; 0.5% BSA, 0.05% Saponin, 0.01% Triton X-100, 0.05% Sodium Azide in 0.1 M Phosphate Buffered Saline (PBS), for 30 min at room temperature and subsequently incubated in primary antibody diluted in blocking buffer at room temperature for 3 days in a humidity chamber using anti-S-protein (mouse, 1:100, in-house antibody SCV-1E8 (Morgan et al., 2023)) in conjunction with either anti-NeuN (chicken, 1:3000, Merck, ABN91), anti-Iba1 (rabbit, 1:1000, Wako, #019–19,741), or anti-GFAP (rabbit, 1:1000, Abcam, ab7260). Slides were washed 4 times in PBS for 15 min. Slides were further incubated in blocking buffer for 5 min prior to adding the species-specific secondary antibody at room temperature overnight in a light-proof humidity chamber: Alexa fluor-546 anti-mouse (1,1,000, Invitrogen, A11030), Alexa fluor-647 anti-rabbit (1,1,000, Invitrogen, A32733), or Alexa fluor-488 anti-chicken (1,1,000, Invitrogen, A11039). Slides were washed once in PBS and incubated in DAPI diluted (1 μg/ ml, Merck) in saline for 5 min. Slides were washed 3x in PBS for 15 min and were mounted with DABCO mounting media.

Sections were imaged using an Olympus UPLXAPO 10x/0.4 NA air objective, 20x/0.8 NA air objective and an UPLXAPO 60x/1.42 NA oil-immersion objective mounted on a spinning disk confocal microscope (SpinSR10; Olympus, Japan) built on an Olympus IX3 body equipped with two ORCA-Fusion BT sCMOS cameras (Hamamatsu Photonics K.K., Japan) and controlled by Olympus cellSens software. Images were acquired as 3D Z-stack tile images and were deconvolved using Huygens Professional Deconvolution Software (Scientific Volume Imaging, Netherlands).

### RNA-Seq and bioinformatics

RNA-Seq was undertaken as described using Illumina Nextseq 550 platform generating 75 bp paired end reads (Rawle et al., 2021; Bishop et al., 2022). The per base sequence quality for >90% bases was above Q30 for all samples. Mouse RNA-Seq reads were aligned to a combined mouse (GRCm39, version M27) and SARS-CoV-2 (Wuhan, NC_045512.2) reference genome using STAR aligner. Organoid RNA-Seq reads were aligned in the same manner except that the human (GRCh38, version 38) reference genome was used. Read counts for host genes and SARS-CoV-2 genomes were generated using RSEM v1.3.1, and differentially expressed genes were determined using EdgeR v3.36.0. To avoid missing type I IFN genes, which have low read counts (Wilson et al., 2017), a low filter of row sum normalized read count >1 was used.

DEGs in direct and indirect interactions were analyzed using Ingenuity Pathway Analysis (IPA, v84978992) (QIAGEN) using the Canonical pathways, Up-Stream Regulators (USR) and Diseases and Functions features as described (Rawle et al., 2022b). Gene Set Enrichment Analysis (GSEAs) were undertaken using GSEA v4.1.0 with gene sets provided in MSigDB (≈ 50,000 gene sets) and in Blood Transcription Modules and log_2_ fold-change-ranked gene lists generated using DESeq2 v1.34.0 as described (Dumenil et al., 2022; Rawle et al., 2022a). Relative abundances of immune cell types in BA.5-infected mouse brains were estimated from RSEM ‘expected counts’ using SpatialDecon v1.4.3 (Danaher et al., 2022) with the ‘ImmuneAtlas_ImmGen_cellFamily’ profile matrix and Pheatmap v1.0.12 in R v4.1.0.

IPA USR cytokine signatures obtained from BA.5-infected K18-hACE2 mouse brains were compared to gene expression data from two studies on COVID-19 patient brains (Fullard et al., 2021; Yang et al., 2021). For the Yang and Fullard studies there were 20 and 45 gene expression data sets, respectively, for the different tissues and cell-types. DEGs sets (*n* = 20 and 45) were derived by applying a *q* < 0.05 filter. These DEG sets were then concatenated to generate a single DEG list for each of the two studies. When a gene was present in more than one DEG set, the mean of the fold changes was used for the concatenated DEG list. IPA USR analysis was then performed as described above.

### Statistics

Statistical analyzes of experimental data were performed using IBM SPSS Statistics for Windows, Version 19.0 (IBM Corp., Armonk, NY, United States). The t-test was used when the difference in variances was <4, skewness was > − 2 and kurtosis was <2. For non-parametric data the Kolmogorov–Smirnov test was used.

## Results

### Omicron BA.5 and XBB are lethal in K18-hACE2 mice

**Figure 1:**
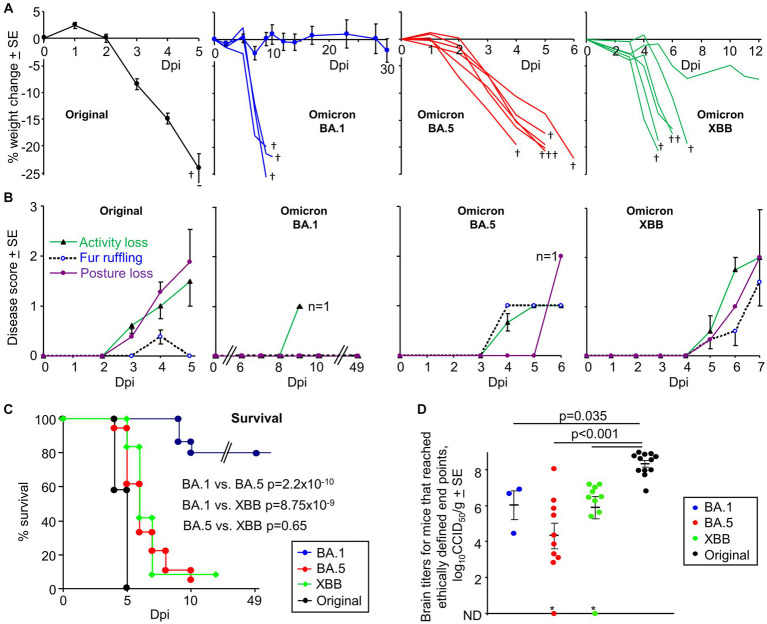
K18-hACE2 mice infected with an original ancestral isolate, omicron BA.1, BA.5 and XBB isolates. **(A)** Percent weight change after intranasal infection with four SARS-CoV-2 isolates dose (5×10^4^ CCID_50_). Original ancestral isolate (*n* = 5–12 mice measured per time point), means and SEs are plotted for all mice (data from 3 independent experiments). BA.1, three mice showed weight loss >20% (requiring euthanasia, †) and are graphed individually; means and SE are plotted for the surviving mice (*n* = 4–15 mice measured per time point). BA.5 (*n* = 6) graphed individually. XBB (*n* = 6) graphed individually. † mice reached ethically defined end points for euthanasia. **(B)** Disease scores for the indicated overt disease symptoms for animals described in “**A**”. For BA.1 no overt symptoms were seen except in a single animal on 9 dpi, with this animal one of the 3 that required euthanasia due to weight loss. For BA.5 the remaining mouse (*n* = 1) on 6 dpi showed a Posture score of 2. For XBB the one surviving mouse recovered after 7 dpi. **(C)** Kaplan Meyer plot showing percent survival; *n* = 12 for original, *n* = 12 for XBB, *n* = 18 for BA.5 and *n* = 24 for BA.1 (data from 2 to 3 independent experiments). Statistics by log-rank tests comparing survival rates. **(D)** Viral tissue titers in brains of mice that had reached ethically defined end points for euthanasia (note that this was at a range of different dpi, see Supplementary Figure S1C). All mice with weight loss requiring euthanasia had detectable viral titers in brain (determined by CCID_50_ assays), with two exceptions (*); although the BA.5 infected mouse emerged to be IHC positive (the XBB infected mice was not tested). Statistics by Kolmogorov–Smirnov tests; titers for the 3 omicron variants were not significantly different from each other. ND – not detected (limit of detection ≈ 2 log_10_CCID_50_/g). (Data from 5 independent experiments).

Infection of K18-hACE2 mice with original ancestral isolates of SARS-CoV-2 is well described and results in weight loss and mortality by ≈ 5 days post infection (dpi) (Amarilla et al., 2021; Kumari et al., 2021; Zheng et al., 2021; Carossino et al., 2022; Dumenil et al., 2022; Yu et al., 2022). We re-illustrate this phenomena herein using an original ancestral isolate (SARS-CoV-2_QLD02_) and K18-hACE2 mice, with the ethically defined end-point of >20% weight loss reached by 4–5 dpi (Figure 1A, Original). An omicron BA.1 isolate (SARS-CoV-2_QIMR01_) was substantially less virulent, with only 20% of mice showing weight loss >20% requiring euthanasia by 9/10 dpi (Figure 1A, Omicron BA.1; Supplementary Figure S1A). The reduced lethality of BA.1 isolates in K18-hACE2 mice is consistent with previous reports (Halfmann et al., 2022; Shuai et al., 2022; Tarres-Freixas et al., 2022; Chen et al., 2023). In contrast, infection of K18-hACE2 mice with an omicron BA.5 isolate (SARS-CoV-2_QIMR03_) or an omicron XBB isolate (SARS-CoV-2_UQ01_) resulted in severe weight loss requiring euthanasia in nearly all mice by 4–7 dpi (Figure 1A, BA.5, XBB).

BA.5 and XBB infected mice also showed more overt disease symptoms than BA.1, with XBB showing disease score comparable with an original strain isolate (Figure 1B). Consistent with previous reports (Seehusen et al., 2022; Bauer et al., 2022b), the majority of BA.1 infected mice showed no symptoms.

Kaplan Meier plots illustrate a highly significant difference between BA.1 vs. BA.5 and BA.1 vs. XBB, with no significant difference between BA.5 and XBB (Figure 1C). Mortality from BA.5 and XBB was delayed when compared with the original ancestral isolate, although the mean delay was ≤2 days (Figure 1C). The results for BA.5 contrast with a recent publication reporting that the reduced pathogenicity of early omicron sub lineages was retained for BA.5 (Uraki et al., 2022).

The brain tissue titers were determined for all mice that reach ethically defined disease endpoints for euthanasia (Figure 1D); there are only 3 data points for BA.1 as 80% of these mice did not reach ethically defined endpoints for euthanasia (Figure 1C), with surviving mice showing no detectable brain infections (Supplementary Figure S1C). Virus was detected in all brain samples, with two exceptions (Figure 1D, indicated by *). Of these, the BA.5-infected mouse brain was subsequently found to be positive by IHC (the XBB-infected mouse was not tested). The brain titers in mice infected with the original strain isolate were significantly higher by ≈ 2 logs than the brain titers in mice infected with the omicron isolates (Figure 1D). Lung titers are shown in Supplementary Figure S1B and are lower in omicron infected mice compared to the original strain isolate, consistent with previous reports (Armando et al., 2022; Natekar et al., 2022; Lee et al., 2023; Ying et al., 2023).

Lethality (generally associated with weight loss requiring euthanasia) in the K18-hACE2 model was previously associated with brain infections (Carossino et al., 2022; Fumagalli et al., 2022), an observation that would thus appear to remain largely true for omicron isolates (Figure 1D). Mice with no detectable brain titers did not reach ethically defined end points for euthanasia (Supplementary Figure S1C). However, our observations are in direct contrast to a previous report showing lethal BA.5 infections in K18-hACE2 mice in the absence of brain infection (Imbiakha et al., 2023). It is perhaps worth noting that in our hands C57BL/6 J mice infected with BA.1, BA.5 or XBB (Shuai et al., 2023) show 100% survival (Supplementary Figure S1D), despite robust lung infections (Shuai et al., 2023). We have not observed overt brain infection in these mice, arguing that C57BL/6 J do not provide a robust neuroinvasion model. Although age has been associated with lethal BA.5 infection in K18-hACE, we saw no significant correlation between age of mouse and lethality (Supplementary Figure S1E).

### Immunohistochemistry of BA.5 and XBB brain infection in K18-hACE2 mice

Given the robust neuroinvasion seen for BA.5 and XBB that was not evident for BA.1 in K18-hACE2 mice, we sought comprehensively to characterize the brain infection and pathology for these emerging variants in this mouse model. The fulminant brain infection seen after infection of K18-hACE2 mice with original ancestral isolates is well described, with widespread infection of neurons in various brain regions, including the cortex (Carossino et al., 2022; Rothan et al., 2022; Seehusen et al., 2022; Vidal et al., 2022; Morgan et al., 2023). A similar pattern of brain infection was observed using our K18-hACE2 mice and an original ancestral isolate, with immunohistochemistry (IHC) undertaken using a recently developed anti-spike monoclonal antibody (SCV2-1E8) (Morgan et al., 2023; Supplementary Figure S2A).

**Figure 2:**
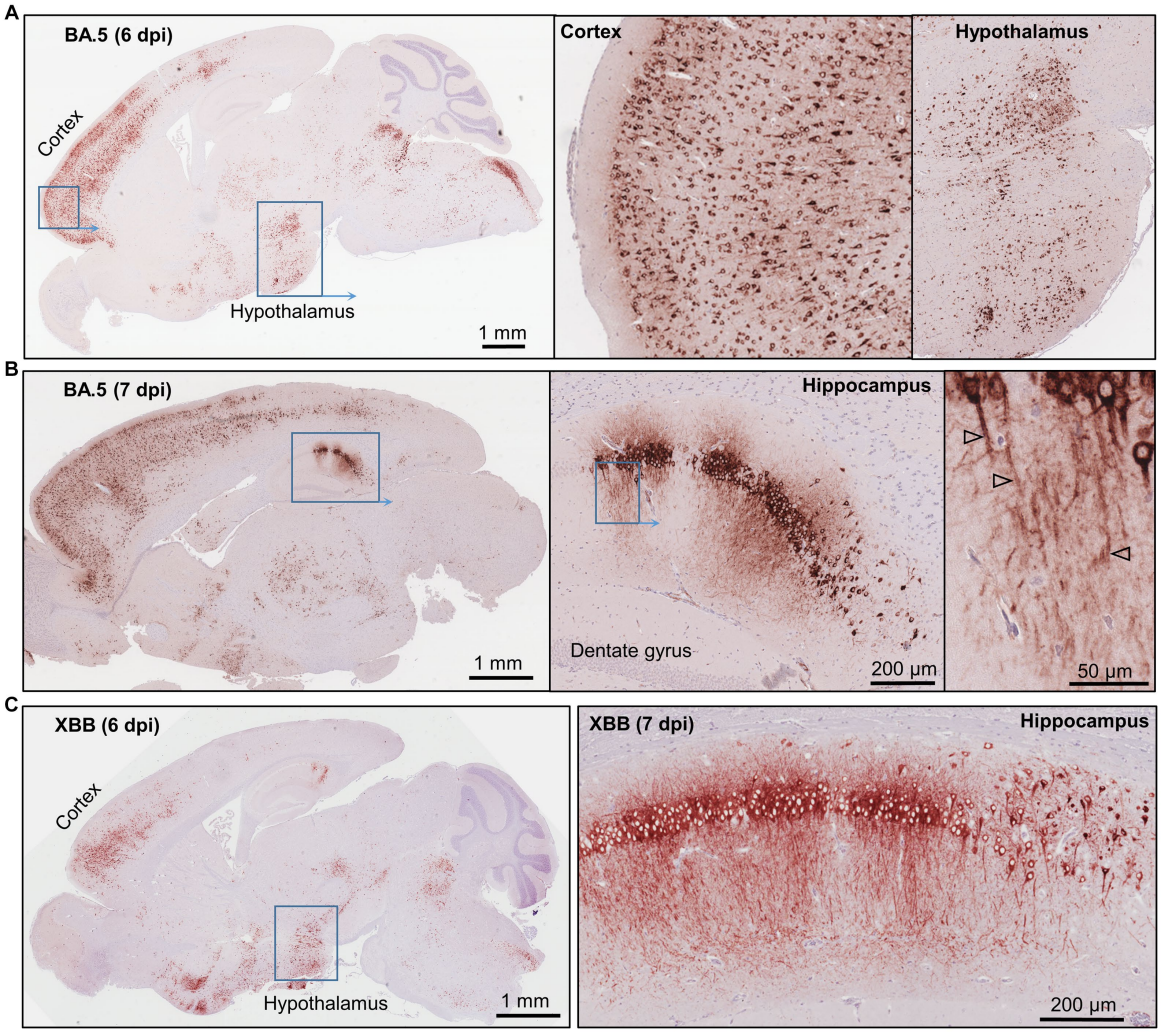
Immunohistochemistry of brains of BA.5 and XBB infected K18-hACE2 mice using an anti-spike monoclonal antibody. **(A)** Brain of BA.5-infected K18-hACE2 mouse 6 dpi showing IHC staining in the cortex and hypothalamus. Insert enlargements on the right. **(B)** As for “a” showing staining of the hippocampus 7 dpi. Far right shows staining of the axons (arrowheads). **(C)** XBB-infected K18-hACE2 mouse showing IHC staining in the cortex and hypothalamus, 6 dpi. Staining of the hippocampus for a mouse euthanized 7 dpi.

IHC staining of brains of K18-hACE2 mice infected with BA.5 or XBB also showed widespread infection of cells in the cortex, as well as the hippocampus and the hypothalamus (Figures 2A–C). Viral RNA and protein have been detected in the cortex (Song et al., 2021; Shen et al., 2022) and hypothalamus (Stein et al., 2022) of post-mortem COVID-patients. Viral protein has also been identified in the hippocampus of such patients (Emmi et al., 2023), with disruption of the hippocampus also reported (Douaud et al., 2022; Radhakrishnan and Kandasamy, 2022). In the hippocampus of K18-hACE2 infected mice, viral antigen could also be clearly seen in dendrites and axons (likely neural) (Figures 2B,C, right hand panels), with viral antigen staining in neurites previously shown in human brain organoids (Bullen et al., 2020).

As described above, brain infection was generally associated with weight loss and mortality in the K18-hACE2 model. Perhaps of note, even the low level of IHC-detectable SARS-CoV-2 infection (Supplementary Figure S2B) seen in one BA.5-infected mouse (Figure 1D, *BA.5) was associated with weight loss that required euthanasia, suggesting that fatal outcomes may not always require a fulminant brain infection.

### Ba.5 infects neurons in K18-hACE2 mice

**Figure 3:**
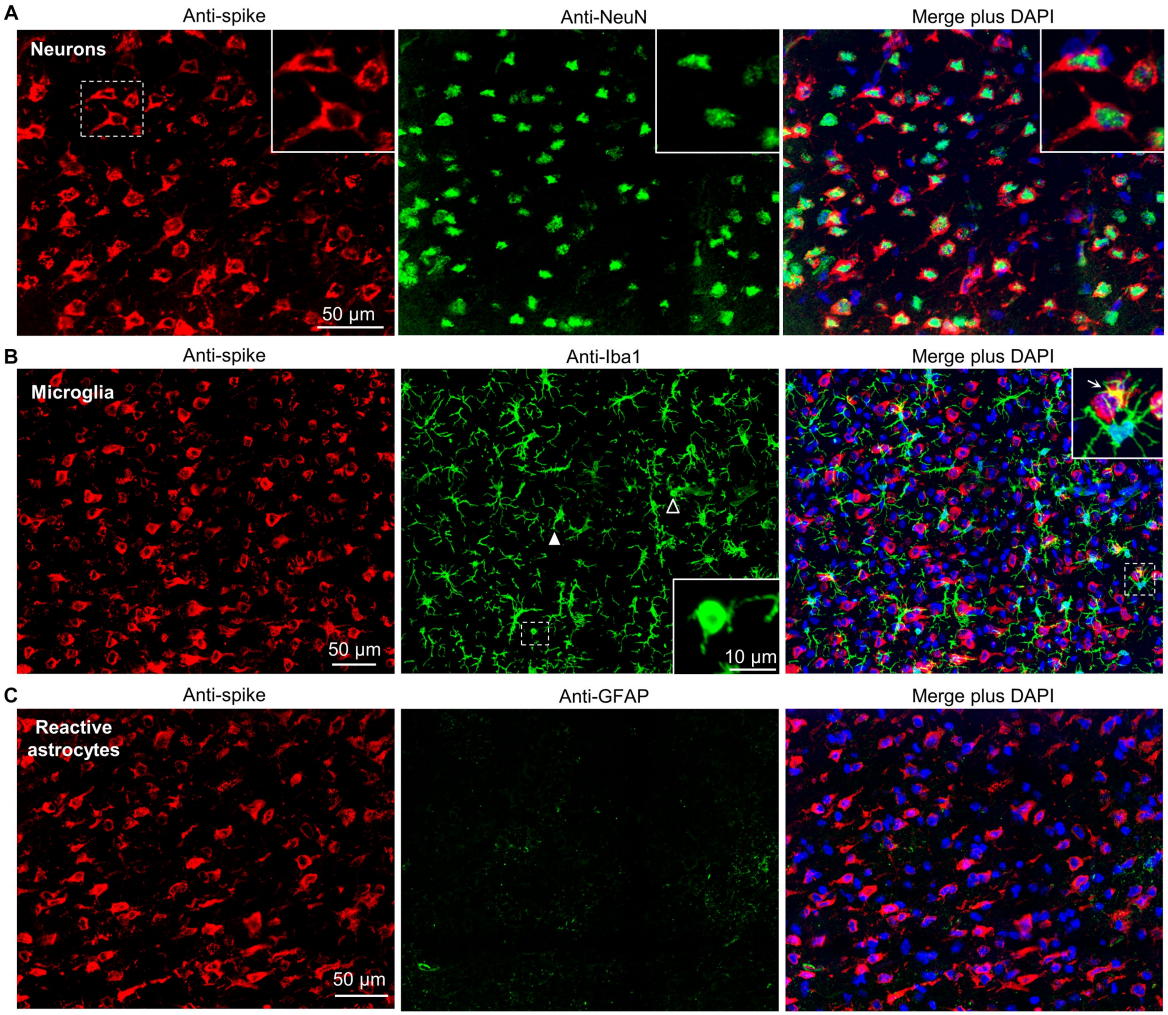
Dual labeling fluorescence immunohistochemistry of BA.5 infected brains from K18-hACE2 mice. **(A)** Sections from formalin fixed and paraffin embedded brains from BA.5 infected K18-hACE2 mouse brains (5 dpi) were analyzed by immunofluorescence. Sections were stained by indirect immunofluorescence with the anti-spike monoclonal antibody (red), and antibodies specific for a neuronal nuclear marker - NeuN (green). Dashed white box (left) indicate cells enlarged in the three inserts (top right). **(B)** As for “a” but costaining with the pan-microglial marker–Iba1 (green). Anti-Iba1 (middle panel); an enlargement of the dashed box (middle panel) is shown in the insert (bottom right), and indicates a microglial cell with amoeboid morphology. Another microglial cell with amoeboid morphology is indicated with a white unfilled arrowhead. White filled arrowhead indicates a microglial cell with bushy morphology. Merged plus DAPI (right panel); an enlargement of the dashed box is shown top right, with arrow indicating yellow (red green overlap), possibly associated with phagocytosis of infected cell debris. **(C)** As for “a” but costaining with a reactive astrocyte maker–GFAP (green).

To confirm infection of neurons by BA.5, the cortex of infected K18-hACE2 mice were co-stained with the anti-spike monoclonal antibody and anti-NeuN, a neuronal nuclear antigen marker. Extensive co-localization within the same cells was observed (Figure 3, Neurons) illustrating that neurons are a primary target of BA.5 infection in K18-hACE2 mouse brains. Co-staining with anti-spike and anti-Iba1, a pan-microglia marker, showed minimal overlap with anti-spike (Figure 3, Microglia), arguing that microglia are not a major target of infection. Occasional overlap (yellow) may be due to phagocytosis of debris from virus-infected cells (Martínez-Mármol et al., 2023). Despite being surrounded by infected neurons, most microglia retained their ramified morphology, although some cells with bushy and amoeboid morphology were present (Figure 3, Microglia) indicating activation-associated retraction of processes (Pinto and Fernandes, 2020). Microglia activation was also indicated by histology and RNA-Seq (see below). Although occasionally seen (Supplementary Figure S3A), anti-GFAP staining was minimal around infected neurons (Figure 3, Reactive astrocytes), arguing that astrocytes are largely not being activated. RNA-Seq also did not identify GFAP as a significantly up-regulated gene, nor did bioinformatics identify an astrocyte activation signature (see below; Supplementary Table S2). The apparent lack of astrocyte activation despite a fulminant SARS-CoV-2 brain infection (at least in mice), with their antiviral and neuroprotective activities thus presumably largely absent, is perhaps perplexing, but is consistent with SARS-CoV-1 studies (Netland et al., 2008).

### Brain lesions identified by H&E in BA.5 and XBB infected K18-hACE2 mice

**Figure 4:**
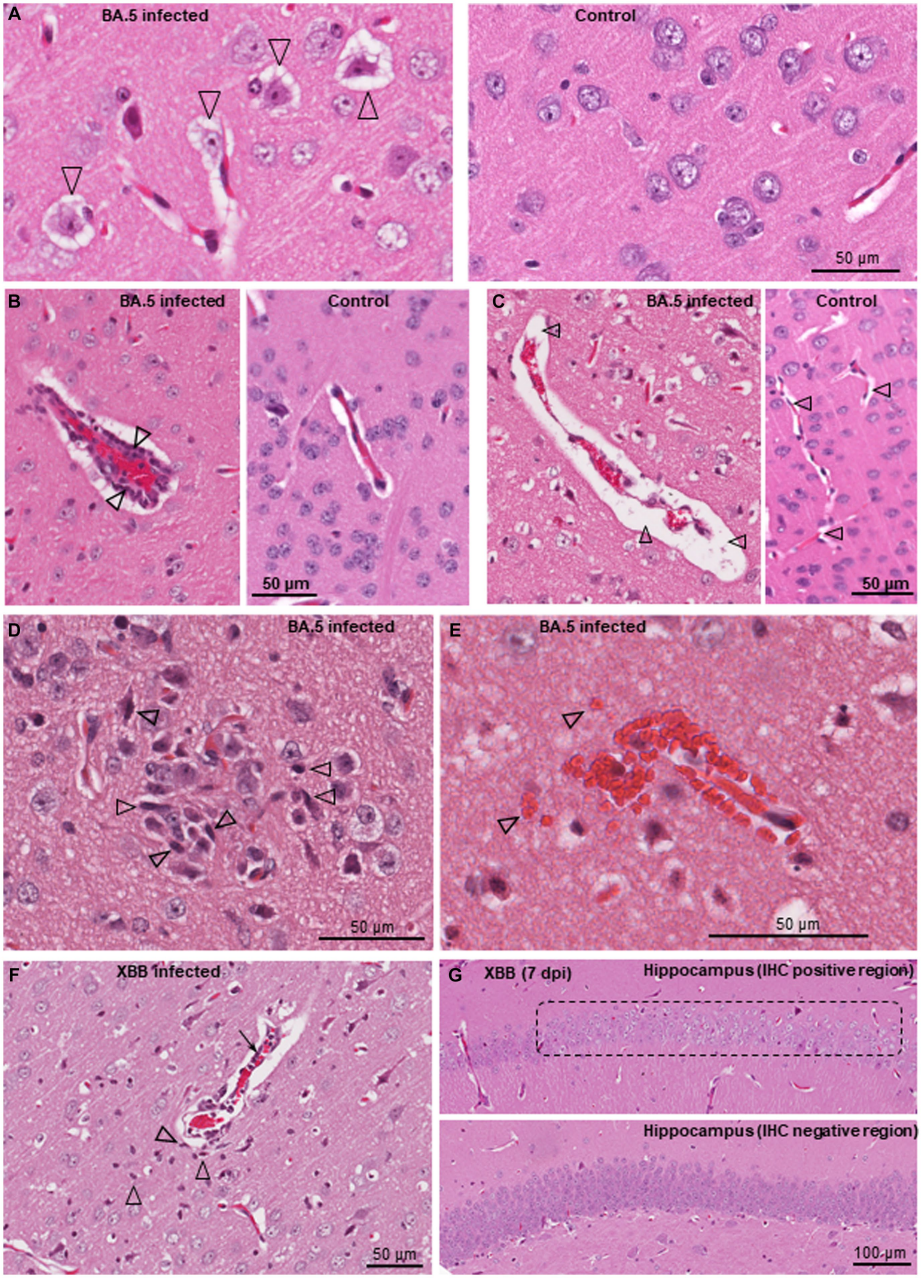
Histological lesions in brains of BA.5 and XBB infected K18-hACE2 mice. H&E staining of brains from BA.5 **(A–E)** and XBB **(F,G)** infected K18-hACE2 mice. **(A)** Neuron vacuolation (hydropic degeneration) of neurons (arrowheads) in the cortex 4 dpi. A control brain from mice inoculated with UV-inactivated BA.5 is shown on the right. **(B)** Perivascular cuffing. A venule (red blood cells in the center) is surrounded by leukocytes (two leukocytes are indicated by arrowheads), 7 dpi. A control venule is shown on the right. **(C)** Focal vasogenic edema; fluid filled perivascular space (arrowheads), 6 dpi. A control is shown on the right, with arrowheads showing normal perivascular spaces. **(D)** Microglial nodule; accumulation of microgliocytes (some typical microgliocytes indicated with arrowheads), 6 dpi. **(E)** Small hemorrhagic lesion, 7 dpi (arrowheads indicate some extravascular red blood cells). **(F)** Lesion in the cortex (from the IHC positive region in Figure 2C, left panel) showing perivascular cuffing (arrow), microgliosis (arrowheads) and vasogenic edema (as in “c”), 6 dpi. **(G)** Loss of hematoxylin staining of neurons in the hippocampus (chromatolysis) in the anti-spike positive region shown by IHC in Figure 2C (right panel). An IHC spike negative region from the hippocampus of the same mouse is shown as a control (bottom image).

The brains of BA.5 and XBB infected K18-hACE2 mice showed a number of histological lesions. Neuron vacuolation (hydropic degeneration) was clearly evident (Figure 4A), and has been reported previously for infection of K18-hACE2 mice with an original ancestral isolate (Vidal et al., 2022), and was also observed in a non-human primate (NHP) model of SARS-CoV-2 infection (Rutkai et al., 2022). The presence of viral antigen in the cortex was associated with apoptosis (Supplementary Figure S3B) and a high intensity of H&E-detectable lesions (primarily vacuolation), but was not associated with local immune cell infiltrates (Supplementary Figure S4). The lack of infiltrates around areas of infection has also been noted in COVID-19 patients (Song et al., 2021). Perivascular cuffing (Figure 4B) is well described in histological examinations of brains from deceased COVID-19 patients (Matschke et al., 2020; Awogbindin et al., 2021; Schwabenland et al., 2021; Rosu et al., 2022; Serrano et al., 2022). Other lesions observed in the BA.5-infected K18-hACE2 mouse brains, that have also been described in post-mortem COVID-19 patients, include perivascular edema (Figure 4C; Maiese et al., 2021; Pajo et al., 2021; Martin et al., 2022), occasional microglial nodules (Figure 4D; Al-Dalahmah et al., 2020; Matschke et al., 2020; Awogbindin et al., 2021; Schwabenland et al., 2021), and occasional small hemorrhagic lesions (Figure 4E; Mukerji and Solomon, 2021; Rosu et al., 2022). XBB infected mouse brains showed very similar lesions (Figure 4F, microgliosis, perivascular cuffing, perivascular edema). Chromatolysis, indicative of injury, was also evident in hippocampal neurons (Figure 4G, dashed box) in the IHC positive region identified in Figure 2C. Despite fulminant brain infection not being a feature of human COVID-19, some histological lesions seen in COVID-19 patient brains are shared with K18-hACE2 mice, although it remains unclear which lesions require direct brain infection.

### RNA-Seq of BA.5-infected K18-hACE2 mouse brains

**Figure 5:**
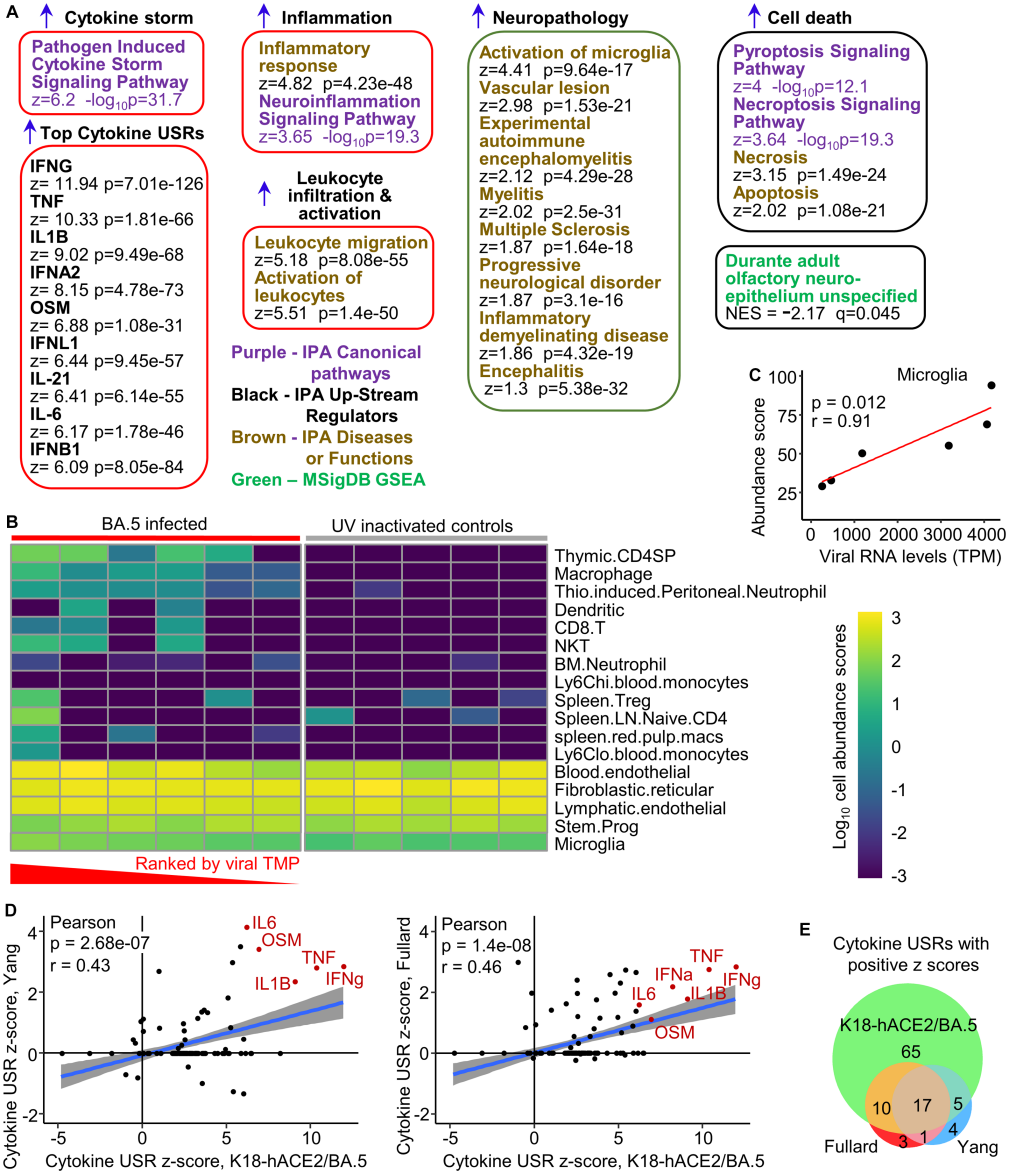
Transcriptome signatures in brains of K18-hACE2 mice infected with BA.5. **(A)** RNA-Seq of BA.5 infected brains (*n* = 6) compared with brains of mice inoculated with UV-inactivated BA.5 (*n* = 5) identified 437 DEGs. The DEGs were analyzed by Ingenuity Pathway Analysis (IPA) and GSEAs using the Molecular Signatures Data Base (MSigDB), with a representative sample of annotations shown and grouped by theme (a full list is provided in Supplementary Table S1). **(B)** The RNA-Seq expression data from brains of BA.5 infected K18-hACE2 mice were analyzed by SpatialDecon to provide estimates of cell type abundances. The BA.5 infected samples were ranked by viral RNA levels (highest to lowest). Cell types were clustered using the complete-linkage of Euclidean distance. **(C)** Relative cell type abundances for Microglia correlates with viral RNA levels. Statistics by Pearson correlation. **(D)** IPA cytokine USRs obtained from brains of BA.5 infected K18-hACE2 mice were compared with IPA cytokine USRs obtained using two DEG lists generated from publically available single-cell RNA-Seq data of selected brain tissues from deceased COVID-19 patients. Where a cytokine USR is identified in human but not mouse (or *vice-versa*), a z-score of zero is given to the latter. **(E)** Venn diagram showing overlaps between the cytokine USRs from the two human (Fullard and Yang) and the BA.5 infected K18-hACE2 mouse study (RNA-Seq data of brain tissues).

Mice were infected as in Figure 1 (BA.5) and euthanized when weight loss reached the ethically defined endpoint of 20% (Supplementary Figure S5A). Control mice received the same inoculation of UV-inactivated BA.5. Brains were examined by RNA-Seq (BioProject ID: PRJNA911424), with the PCA plot shown in Supplementary Figure S5B and viral RNA levels in Supplementary Figure S5C. Differentially expressed genes (DEGs) (*q* < 0.05, *n* = 437) were analyzed by Ingenuity Pathway Analysis (IPA) (Bishop et al., 2022; Dumenil et al., 2022; Supplementary Table S2). Selected representative IPA annotations, grouped by themes, are shown in Figure 5A. The dominant annotations illustrate a cytokine storm, with the top cytokine Up Stream Regulators (USRs) including interferons both type II (IFNγ) and type I (IFNα2, IFNλ1 IFNβ1), as well as TNF, IL-1 and IL-6, all previously well described for SARS-CoV-2 infections (Bishop et al., 2022). The concordance for cytokine USRs for brain and lung infection, and for the three virus isolates, was high (Supplementary Figure S5D), arguing that inflammatory responses are generally very similar for brain and lungs and for the different SARS-CoV-2 variants.

A large series of annotations were associated with leukocyte migration and activation (Supplementary Table S2), with the top two overarching annotations shown (Figure 5A, Leukocyte migration, Activation of leukocytes). These annotations are consistent with the perivascular cuffing seen by H&E (Figure 4B). An additional series of neuropathology-associated annotations were also identified with high z-scores and significance (Figure 5A, Neuropathology). Activation of microglia and vascular lesions (Figure 5A) were consistent with the histological findings (Figures 4B–D). Apoptosis of neurons was reported in the NHP model (Rutkai et al., 2022), with pyroptosis in the CNS of COVID-19 patients also proposed (Sepehrinezhad et al., 2021). Gene Set Enrichment Analyzes (GSEAs) using gene sets provided in MSigDB (≈ 50,000 gene sets) and in Blood Transcription Modules, generated broadly comparable results to those obtained from IPA (Supplementary Table S2). In addition, a significant negative enrichment (negative NES) for olfactory neuroepithelium genes (MSigDB) (Durante et al., 2020) was also identified (Figure 5A), suggesting loss of cells in this tissue in BA.5-infected mouse brains. Infection of the olfactory epithelium likely provides the entry route into the brain in this model (Dumenil et al., 2022; Fumagalli et al., 2022).

To provide insights into the nature of the leukocyte infiltrates, cell type abundance estimates were obtained from the RNA-Seq expression data using SpatialDecon (Danaher et al., 2022; Figure 5B). The inflammatory infiltrate appeared primarily to comprise immature CD4 T cells (Hosseinzadeh and Goldschneider, 1993), macrophages, neutrophils, dendritic cells, CD8 T cells and NKT cells, with increased cell abundance scores seen with increasing viral RNA levels (Figure 5B, TPM - transcripts per million; Supplementary Figure S5E). Although not substantial, increased cell abundance scores also increased with viral RNA levels for microglia (Figure 5C).

In summary, the bioinformatic analyses illustrate that the inflammatory responses in BA.5-infected K18-hACE2 mouse brains are largely innate (4–6 dpi) and typical of acute SARS-CoV-2 infections, with many annotations consistent with histological findings.

### Some concordance in cytokine gene expression patterns between brains of severe COVID-19 patients and brains from SARS-CoV-2 infected K18-hACE2 mice

We previously illustrated that inflammatory pathways identified by RNA-Seq of lungs from COVID-19 patients showed highly significant concordances with SARS-CoV-2 infected lungs from K18-hACE2 mice (Bishop et al., 2022). Two single-cell RNA-Seq data sets are publically available for selected human brain tissues (choroid plexus, medulla oblongata, and pre-frontal cortex) from deceased COVID-19 patients (Fullard et al., 2021; Yang et al., 2021). DEG sets from each tissue and cell-type were concatenated to create one overall DEG list for each of the two human studies. These DEG lists were analyzed by IPA as above, and the cytokine USR z-scores compared with those obtained from brains of BA.5-infected K18-hACE2 mice. Significant correlations emerged for both human studies (Figure 5D), with many of the prominent cytokines associated with SARS-CoV-2 infections (Bishop et al., 2022) identified in both species (Figure 5D, cytokines shown in red text). Overall, 80% of the cytokine USRs identified in humans also identified in K18-hACE2 mice (Figure 5E). However, the fulminant lethal brain infection likely explains the higher number of USRs identified for brains of K18-hACE2 mice (65 out of 97) that are not seen in COVID-19 patient brains (Figure 5E).

Gene Set Enrichment Analyzes (GSEAs) also illustrated that DEGs up-regulated in brains of COVID-19 patients (log_2_ fold change >1) (Fullard et al., 2021; Yang et al., 2021), were significantly enriched in the ranked gene list from brains of BA.5-infected K18-hACE2 mice (Supplementary Figure S6).

Thus both at the pathway level and the gene expression level, a level of concordance was apparent between cytokine mRNA expression data from (i) brain tissues of severe COVID-19 patients and (ii) brains of BA.5-infected K18-hACE2 mice.

### Infection of human cortical brain organoids

**Figure 6:**
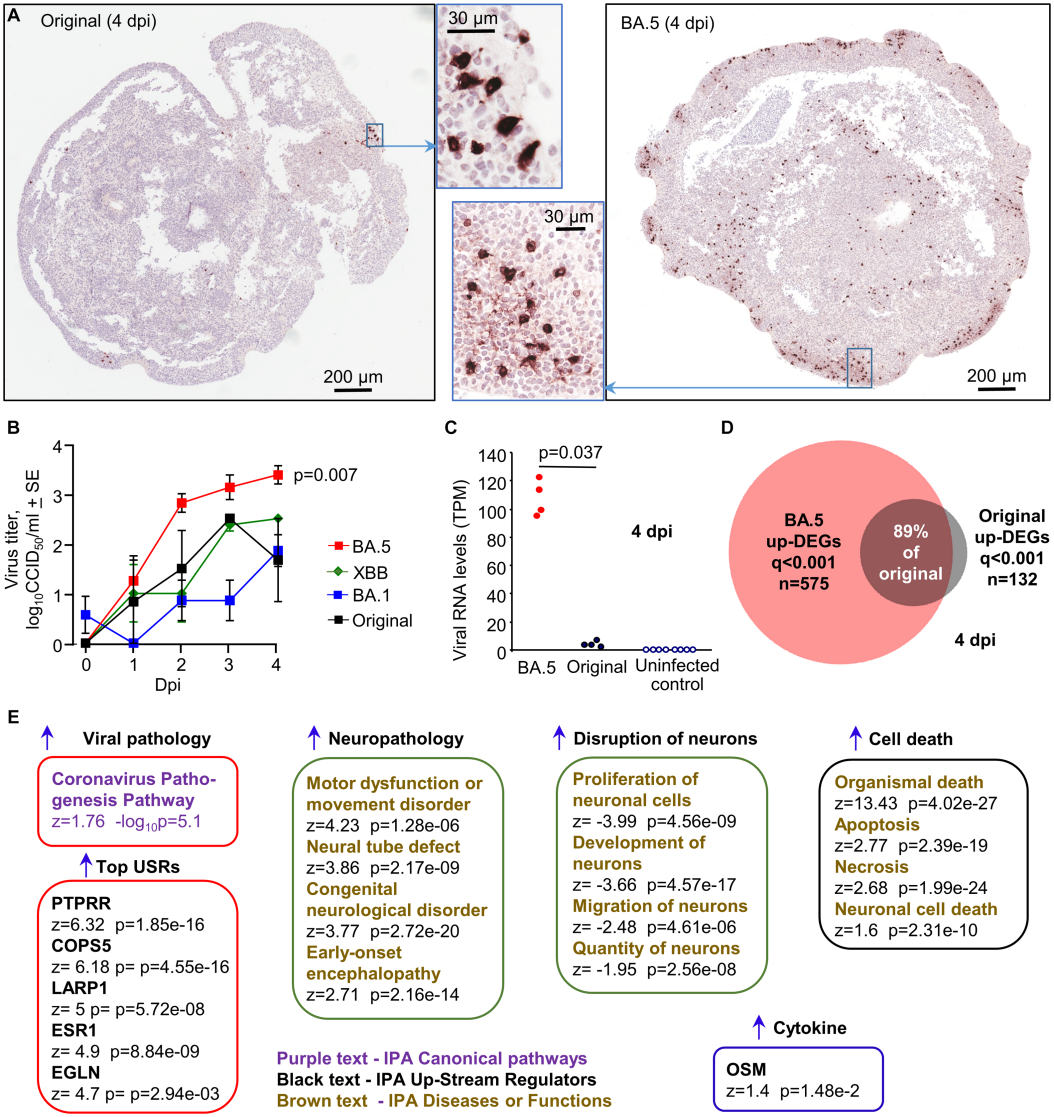
Infection of cortical brain organoids with original ancestral, BA. 1, BA.5 and XBB. **(A)** IHC of brain organoids 4 dpi with original ancestral and BA.5 using an anti-spike monoclonal antibody. IHC staining for BA.1 and XBB infected organoids is shown in Supplementary Figures S9A,B. **(B)** Viral titers in the supernatant of the organoid cultures sampled at the indicated dpi. Data is derived from two independent experiments. Titers for BA.5 (*n* = 8) vs. BA.1 (*n* = 7) were significantly different (*p* = 0.007) on days 2, 3 and 4. Titers for XBB (*n* = 4) vs. BA.1 (*n* = 7) were significantly different (*p* = 0.009) when the data for 3 and 4 dpi were combined. Statistics by Kolmogorov–Smirnov tests. **(C)** RNA-Seq-derived viral reads counts for infected organoids, 4 dpi. **(D)** Venn diagram showing overlap of up-regulated DEGs for organoids infected with BA.5 and original ancestral. **(E)** DEGs (*n* = 2,390, *q* < 0.001) generated from RNA-Seq of BA.5 infected organoids (*n* = 4) 4 dpi vs. uninfected organoids (*n* = 4) were analyzed by IPA. Selected and representative annotations are shown (for full data set see Supplementary Table S3).

Human, induced pluripotent cells (hiPSCs), derived from a primary dermal fibroblast line (HDFa) from a normal human adult, were used to generate approximately spherical, ≈ 2–3 mm diameter, “mini-brains” using a rotating incubator (Supplementary Figure S7A). RNA-Seq and IHC illustrated that 30 day old organoids were comprised primarily of neural progenitor cells (expressing SOX2 and nestin) and immature neurons expressing MAP2 (Microtubule-Associated Protein 2) and TUBB3 (tubulin beta 3; Supplementary Figures S7B,C). Such organoids were infected with the XBB, BA.5, BA.1 and the original ancestral isolate (MOI ≈ 1) and were cultured for 4 days. Dual labeling fluorescent IHC illustrated that BA.5 infected MAP2-negative cells, and some MAP2-positive cells (Supplementary Figure S8). The BA.5 virus infected substantially more cells in the organoids than the original ancestral (Figure 6A) or the BA.1 viruses (Supplementary Figure S9A). XBB also infected slightly more cells than BA.1 (Supplementary Figure S9B). The small area infected with the original ancestral isolate (Figure 6A, Original, insert) corresponded to an area of the organoid with IHC-detectable anti-hACE2 staining (Supplementary Figure S9C). The overall expression of hACE2 mRNA was low, with transmembrane protease serine 2 (TMPRSS2) mRNA often undetectable (Supplementary Figure S9D). Viral titers in the supernatants of the organoid cultures increased over the 4 day period, with BA.5 titers significantly higher than BA.1 titers by 1–2 logs (Figure 6B, *p* = 0.007, 2, 3 and 4 dpi). XBB titers were also up to ≈1 log higher, which reached significance if data from 4 and 5 dpi were combined (Figure 6B, *p* = 0.009, 4 & 5 dpi). RNA-Seq of organoids harvested 4 dpi also illustrated that viral RNA levels were ≈ 25 fold higher for organoids infected with BA.5 than those infected with an original ancestral isolate (Figure 6C).

RNA-Seq of BA.5 infected human cortical brain organoids (4 dpi) compared with uninfected organoids provided 2,390 DEGs (*q* < 0.001), of which 575 were up-regulated genes (Supplementary Table S3). RNA-Seq of original ancestral isolate-infected organoids provided 252 DEGs (*q* < 0.001), of which 132 were up-regulated (Supplementary Table S3). Of the 132 up-regulated DEGs, 118 were also identified in the BA.5 infected organoids (Figure 6D), arguing that the original ancestral isolate is not inducing fundamentally different response in these organoids, and that the increased number of DEGs for BA.5 is likely due to more robust infection.

The 2,390 DEGs for BA.5 were analyzed by IPA (Supplementary Table S3), with “Coronavirus Pathogenesis Pathway” identified as a top canonical pathway (Figure 6E). The top USRs were (i) PTPRR (a protein tyrosine phosphatase receptor), which was recently identified in a study of brains from SARS-CoV-2 infected hamsters and is associated with depression in humans (Serafini et al., 2022), (ii) COPS5 (COP9 signalosome subunit 5), whose mRNA is bound by SARS-CoV-2 NSP9, perhaps resulting in suppression of host responses (Banerjee et al., 2020), (iii) LARP1, a translational repressor that binds SARS-CoV-2 RNA (Schmidt et al., 2021), (iv) ESR1 (nuclear estrogen receptor), which is important for ACE2 expression (Oner et al., 2022), (v) EGLN1, oxygen sensors that target HIF α subunits for degradation, with HIF-1α promoting SARS-CoV-2 infection and inflammation (Tian et al., 2021). IPA Diseases and Functions feature identified a series of neuropathology-associated annotations, including a motor dysfunction signature, with motor deficits documented for severe COVID-19 patients (Graham et al., 2022). Consistent with the IHC data, a series of signatures describe disruption and death of neurons (Figure 6E). No significant up-regulation of classical inflammation or IFN signatures were identified, with the possible exception of oncostatin M (OSM) (Figure 6E). Serum concentrations of this IL-6 family pleiotropic cytokine show a strong positive correlation with COVID-19 severity (Arunachalam et al., 2020). However, OSM can also be secreted by neural cells, but in the brain it is thought often to play a neuroprotective role (Houben et al., 2019).

## Discussion

We illustrate herein that the BA.5 and XBB variants show greater propensities to enter the brain and infect neurons in K18-hACE2 mice when compared to BA.1. In addition, BA.5 showed an increased capacity to infect human brain organoids. Taken together these results argue that these two more recent omicron variants of concern may have enhanced neurotropic properties when compared to an earlier omicron variant in these models.

The increased infection of brains by BA.5 and XBB over BA.1 in K18-hACE2 mice may be associated with the enhanced fusion activity of the later omicron variants (Tamura et al., 2023; Tang et al., 2023), which is usually associated with an enhanced ability to utilize TMPRSS2 and/or increased binding affinity for ACE2 (Aggarwal et al., 2022; Tamura et al., 2023). TMPRSS2 utilization is associated with virulence (Abbasi et al., 2021), even for omicron variants (Iwata-Yoshikawa et al., 2022), and is involved in neurotropism in K18-hACE2 mice (Li et al., 2021). An increased ability to infect, not just the TMPRSS2-positive sustentacular cells, but also TMPRSS2-low cells in the murine olfactory epithelium (Fodoulian et al., 2020), may thus promote entry of BA.5 and XBB into the brains of K18-hACE2 mice when compared with BA.1. In contrast to early omicron variants (Meng et al., 2022; Zhao et al., 2022; Qu et al., 2023), original ancestral isolates show a preference for TMPRSS2 utilization, and rapid fulminant infection of K18-hACE2 brains by such viruses is well described (Rothan et al., 2022; Seehusen et al., 2022). Interestingly, viral sequences from the brains of K18-hACE2 mice and hamsters infected with original ancestral isolates show loss of functional furin cleavage sites (Supplementary Figure S10). TMPRSS2 mRNA expression levels in K18-hACE2 mouse brains are very low (Supplementary Table S2), so TMPRSS2-independent infection (Qu et al., 2023) would likely be selected as the virus spreads within the brains. Such furin cleavage site deletions were not seen in BA.1 or BA.5 sequences from brains of K18-hACE2 mice, likely because omicron viruses can already effectively use the endosomal pathway (Meng et al., 2022; Zhao et al., 2022; Qu et al., 2023). In summary, more efficient use of TMPRSS2-dependent infection by BA.5 and XBB (and original ancestral isolates) compared to BA.1, may promote entry into the brain of K18-hACE2 mice via the olfactory epithelium. Once in the brain, utilization of the endosomal pathway by omicron viruses (and original ancestral isolates with non-functional furin cleavage sites) allows a spreading infection in TMPRSS2-low brain cells (Figure 1D, Brains).

Infection of brain organoids represents a measure of neurovirulence, rather than neuroinvasiveness, as access to cells in this *in vitro* system clearly does not require transit across the cribriform plate (Jiao et al., 2021; Dumenil et al., 2022; De Melo et al., 2023). TMPRSS2 mRNA expression was even lower in the organoids (Supplementary Figure S9D) than in K18-hACE2 brains, with infection of human neurons shown to be TMPRSS2-independent (Kettunen et al., 2023). This is consistent with the poor infection of organoids by the original ancestral isolate (Figures 6A–C). BA.5 would thus appear to have an increased capacity for infection of brain organoids via a TMPRSS2-independent mechanism. This is not due to acquisition of hACE2-independent infection capabilities (Yan et al., 2022; Supplementary Figure S11). Nor is this likely due to an increased ability of BA.5 to counter type I IFN activities (Guo et al., 2022), as such responses were not detected (Figure 6E). hACE2 expression is low in the organoids, suggesting an increased affinity for hACE2 might promote BA.5 infection, with barely detectable levels of hACE2 able to support infection (Rawle et al., 2021). There are a number of differences in the spike protein between the BA.1 and BA.5 isolates including 9 amino acid changes in the receptor binding domain (Supplementary Figure S12), with BA.5 affinity for hACE2 reported as slightly higher in two studies (Tuekprakhon et al., 2022; Wang et al., 2022), but unchanged in a third (Cao et al., 2022). More efficient use by BA.5/XBB of co-receptors such as neuropilin (Cantuti-Castelvetri et al., 2020; Kong et al., 2022) or heparin sulphate proteoglycans (Guimond et al., 2022) might also be involved. Non-spike changes may also play a role (Chen et al., 2023).

To what extent are the observations herein relevant to human disease? Controversy remains regarding how useful the K18-hACE2 mouse models is for understanding human disease, although a considerable body of literature argues that many aspects of respiratory COVID-19 are recapitulated in this model (Yinda et al., 2021; Zheng et al., 2021; Bishop et al., 2022; Park et al., 2022; Ye et al., 2023). The fulminant lethal brain infection in K18-hACE2 mice is clearly not a feature of COVID-19. However, low level brain infections have been reported in hamster (Zhang et al., 2021; De Melo et al., 2023) and primate models (Beckman et al., 2022; Rutkai et al., 2022; Hailong et al., 2023), as well as in a range of human studies, including studies on long-COVID patients (Proal et al., 2023; Supplementary Table S1). The data herein also illustrates that certain features of the infected K18-hACE2 brains are also observed in some patients with severe COVID-19. As confirmed herein for BA.5 and XBB isolates, human neurons can be readily infected *in vitro* (Bullen et al., 2020; Ramani et al., 2020; Song et al., 2021; Hou et al., 2022; Mesci et al., 2022; Shen et al., 2022), with infection of neurons also seen in some COVID-19 patients (Song et al., 2021; Shen et al., 2022; Emmi et al., 2023), in a hamster model (De Melo et al., 2023) and in K18-hACE2 mice (Rothan et al., 2022; Seehusen et al., 2022), with BA.5 infection of neurons shown in Figure 3. A well described neurological manifestation of COVID-19 is anosmia, with infection of the olfactory epithelium implicated in humans (Khurana and Singh, 2022; Ziuzia-Januszewska and Januszewski, 2022), hamsters (De Melo et al., 2023) and in K18-hACE2 mice (Zheng et al., 2021). The olfactory epithelium may also provide access of virus to the brain (Fodoulian et al., 2020; Jiao et al., 2021; Dumenil et al., 2022), with a recent hamster study suggesting SARS-CoV-2 can travel along axons into the olfactory bulb (De Melo et al., 2023). SARS-CoV-2 may also access the brain via a breach in the blood brain barrier (Zhang et al., 2021); however, the lack of viremia (Yinda et al., 2021), and the ability to avoid brain infection by aerosolized delivery of virus into lungs, argues against this route of entry in the K18-hACE2 model (Dumenil et al., 2022; Fumagalli et al., 2022).

Brain infection is not a common feature of COVID-19, but low level infection does appear to manifest in a small group of COVID-19 and long-COVID patients (Supplementary Table S1; Proal et al., 2023). What comorbidities, injuries and/or other factors predispose to brain infection remain unclear. Perhaps of note, anosmia was recently closely linked to long-lasting cognitive problems in COVID-19 patients (RADC, 2022), with anosmia also predicting memory impairment in post-COVID-19 syndrome patients in a separate study (Ruggeri et al., 2023). A higher proportion of patients infected with BA.5 develop anosmia, when compared with BA.1 (Hansen et al., 2023) perhaps due to more rapid and fulminant infection of the upper respiratory tract (Carabelli et al., 2023). Taken together with the data presented herein, BA.5 and XBB may thus show increased risk of acute and long-term neurological complications over earlier omicron variants (Okrzeja et al., 2023).

## Data availability statement

All data is provided in the manuscript and accompanying Supplementary material. Raw sequencing data (fastq files) generated for this publication for RNA-Seq have been deposited in the NCBI SRA, BioProject: PRJNA813692 and are publicly available.

## Ethics statement

The studies involving humans were approved by QIMR Berghofer Medical Research Institute Human Research Ethics Committee and the University of Queensland HREC. The studies were conducted in accordance with the local legislation and institutional requirements. The participants provided their written informed consent to participate in this study. The animal study was approved by QIMR Berghofer MRI Animal Ethics Committee. The study was conducted in accordance with the local legislation and institutional requirements.

## Author contributions

RS: Methodology, Writing – review & editing, Investigation. KY: Investigation, Formal analysis, Writing – review & editing. SE: Investigation, Formal analysis, Visualization, Writing – review & editing. CB: Formal analysis, Visualization, Writing – review & editing. TD: Formal analysis, Writing – review & editing. BT: Investigation, Writing – review & editing. WN: Investigation, Writing – review & editing. TL: Formal analysis, Writing – review & editing. RP: Resources, Writing – review & editing. JS: Resources, Writing – review & editing. AK: Resources, Writing – review & editing. RKS: Formal analysis, Writing – review & editing. ML: Investigation, Writing – review & editing. FM: Formal analysis, Methodology, Supervision, Writing – review & editing, Visualization. DR: Formal analysis, Methodology, Supervision, Writing – review & editing, Project administration. AS: Formal analysis, Methodology, Supervision, Writing – review & editing, Conceptualization, Data curation, Funding acquisition, Project administration, Writing – original draft.
