# Supplementary material for: SARS-CoV-2 omicron BA.5 and XBB variants have increased neurotropic potential over BA.1 in K18-hACE2 mice and human brain organoids
Source: Front Microbiol. 2023 Nov 23;14:1320856. doi: 10.3389/fmicb.2023.1320856 (PMC10706942; doi:10.3389/fmicb.2023.1320856)
Supplement: Supplementary file 2 [file Table_1.DOCX]

**Supplementary Table 1. Summary of Eevidence for SARS-CoV-2 infection of human brain**

| **Samples and References** | **Virus detection method** | **Findings** |
| --- | --- | --- |
| Post-mortem brain samples from 11 COVID-19 patients [1] | ddPCR  ISH  IHC  Virus isolation | 10/11 positive for SARS-CoV-2 RNA by ddPCR and ISH.  CNS neurons positive for SARS-CoV-2 N protein by IHC. Replication competent virus isolated from thalamus (1 patient). |
| 26 brain tissue samples (orbitofrontal cortex sampled via endonasal transethmoidal access) [2] | RT-qPCR  IHC | 5/26 positive for SARS-CoV-2 RNA by RT-qPCR.  CNS astrocytes and neurons positive for SARS-CoV-2 S protein by IHC. |
| Post-mortem brain samples from 17 COVID-19 patients [3] | ISH  IHC | 17 brains positive for SARS-CoV-2 RNA by ISH and S/N protein by IHC. |
| Post-mortem brain samples from 3 COVID-19 patients [4] | IHC | All 3 brains contained staining for SARS-CoV-2 S protein by IHC. |
| Post-mortem brain samples from 40 COVID-19 patients [5] | RT-qPCR  IHC | 21/40 positive for SARS-CoV-2 RNA by RT-PCR.  8 also positive for SARS-CoV-2 N protein by IHC. |
| Post-mortem brain samples from 20 COVID-19 patients (16 brain regions sampled per patient = 320 total samples) [6] | RT-qPCR | 8/21 positive for SARS-CoV-2 RNA by RT-qPCR (Cts, 28.26, 26.37, 35.07, 37.91, 36.75, 38.15, 33.39, 29.46). |
| CNS samples from 33 humans (n=31 olfactory bulb, n=7 olfactory tubercle, n=22 trigeminal ganglion, n=31 medulla oblongata, n=24 cerebellum [7] | RT-PCR  IHC | 10/33 positive for SARS-CoV-2 RNA by RT-qPCR.  4/33 positive for SARS-CoV-2 S protein by IHC. |
| 1 x human brain (sections) [8] | TEM | 80-110 nm viral particles observed in CNS (frontal lobe sections) of SARS-CoV-2 positive patient. |
| 1 x human brain (sections) [9] | TEM | Spherical particles (≈98 nm) with crown shape consistent with SARS-CoV-2 in autopsy sample from severe COVID-19 case |
| 1 x human brain (sections) [10] | TEM | Viral particles at the endothelial neural cell interface and in the cytoplasm of neural cell |
| Post-mortem brain samples from 24 COVID-19 patients [11] | RT-qPCR  IHC | 10/24 positive for SARS-CoV-2 RNA by RT-qPCR (Cts 33-38).  7/24 positive for SARS-CoV-2 S and N proteins by IHC (7 positive in parenchyma, 5 positive within neurons). |
| Human CSF (>30 samples from various studies [12-21] | RT-PCR  ELISA | CSF positive for SARS-CoV-2 RNA (Ct reported in 8 cases; 20, 38, 35.7, 29, 34, 19.5, 37, 37).  3 patients with encephalopathy and encephalitis had anti-SARS-CoV-2 (spike or envelope protein) IgM antibodies in CSF |

CSF = Cerebrospinal Fluid. CNS = Central Nervous System. IHC = Immunohistochemistry. ISH = in-situ hybridization
RT-PCR = Reverse transcription-polymerase chain reaction. RT-qPCR = Reverse transcription-quantitative polymerase chain reaction. TEM = Transmission Electron Microscopy. ELISA = enzyme-linked immunosorbent assay. ddPCR = digital droplet PCR. Ct = cycle threshold (in qPCR)

**References**

1. Stein SR, Ramelli SC, Grazioli A, Chung J-Y, Singh M, Yinda CK, et al. SARS-CoV-2 infection and persistence in the human body and brain at autopsy. Nature. 2022;612:758–63. doi: 10.1038/s41586-022-05542-y.

2. Crunfli F, Carregari VC, Veras FP, Silva LS, Nogueira MH, Antunes A, et al. Morphological, cellular, and molecular basis of brain infection in COVID-19 patients. Proc Natl Acad Sci U S A. 2022;119(35):e2200960119. Epub 2022/08/12. doi: 10.1073/pnas.2200960119. PubMed PMID: 35951647; PubMed Central PMCID: PMCPMC9436354.

3. Shen WB, Logue J, Yang P, Baracco L, Elahi M, Reece EA, et al. SARS-CoV-2 invades cognitive centers of the brain and induces Alzheimer's-like neuropathology. bioRxiv. 2022. Epub 2022/02/09. doi: 10.1101/2022.01.31.478476. PubMed PMID: 35132414; PubMed Central PMCID: PMCPMC8820661.

4. Song E, Zhang C, Israelow B, Lu-Culligan A, Prado AV, Skriabine S, et al. Neuroinvasion of SARS-CoV-2 in human and mouse brain. J Exp Med. 2021;218(3):e20202135. Epub 2021/01/13. doi: 10.1084/jem.20202135. PubMed PMID: 33433624; PubMed Central PMCID: PMCPMC7808299 submitted work; and reported, "AI Therapeutics is currently sponsoring a clinical trial for a therapeutic, which has no relevance for this study, in COVID-19. I am the Chief Scientific Advisor to AI Therapeutics." C.B. Wilen reported personal fees from ZymoResearch outside the submitted work; in addition, C.B. Wilen had a patent for compounds and compositions for treating, ameliorating, and/or preventing SARS-CoV-2 infection and/or complications thereof pending. S. Haik reported a patent to Method for treating prion diseases (PCT/EP 2019/070457) pending. A. Iwasaki reported "other" from RIGImmune and grants from Spring Discovery during the conduct of the study; in addition, A. Iwasaki had a patent to 14/776,463 pending, a patent for a T cell-based immunotherapy for central nervous system viral infections and tumors pending, and a patent to manipulation of meningeal lymphatic vasculature for brain and CNS tumor therapy pending. No other disclosures were reported.

5. Matschke J, Lutgehetmann M, Hagel C, Sperhake JP, Schroder AS, Edler C, et al. Neuropathology of patients with COVID-19 in Germany: a post-mortem case series. Lancet Neurol. 2020;19(11):919-29. Epub 2020/10/09. doi: 10.1016/S1474-4422(20)30308-2. PubMed PMID: 33031735; PubMed Central PMCID: PMCPMC7535629.

6. Serrano GE, Walker JE, Tremblay C, Piras IS, Huentelman MJ, Belden CM, et al. SARS-CoV-2 Brain Regional Detection, Histopathology, Gene Expression, and Immunomodulatory Changes in Decedents with COVID-19. J Neuropathol Exp Neurol. 2022;81(9):666-95. Epub 2022/07/13. doi: 10.1093/jnen/nlac056. PubMed PMID: 35818336; PubMed Central PMCID: PMCPMC9278252.

7. Meinhardt J, Radke J, Dittmayer C, Franz J, Thomas C, Mothes R, et al. Olfactory transmucosal SARS-CoV-2 invasion as a port of central nervous system entry in individuals with COVID-19. Nat Neurosci. 2021;24(2):168-75. Epub 2020/12/02. doi: 10.1038/s41593-020-00758-5. PubMed PMID: 33257876.

8. Paniz-Mondolfi A, Bryce C, Grimes Z, Gordon RE, Reidy J, Lednicky J, et al. Central nervous system involvement by severe acute respiratory syndrome coronavirus-2 (SARS-CoV-2). J Med Virol. 2020;92(7):699-702. Epub 2020/04/22. doi: 10.1002/jmv.25915. PubMed PMID: 32314810; PubMed Central PMCID: PMCPMC7264598.

9. Bulfamante G, Chiumello D, Canevini MP, Priori A, Mazzanti M, Centanni S, et al. First ultrastructural autoptic findings of SARS -Cov-2 in olfactory pathways and brainstem. Minerva Anestesiol. 2020;86(6):678-9. Epub 2020/05/14. doi: 10.23736/S0375-9393.20.14772-2. PubMed PMID: 32401000.

10. Paniz-Mondolfi A, Bryce C, Grimes Z, Gordon RE, Reidy J, Lednicky J, et al. Central nervous system involvement by severe acute respiratory syndrome coronavirus-2 (SARS-CoV-2). Journal of Medical Virology. 2020;92(7):699-702. doi: <https://doi.org/10.1002/jmv.25915>.

11. Emmi A, Rizzo S, Barzon L, Sandre M, Carturan E, Sinigaglia A, et al. Detection of SARS-CoV-2 viral proteins and genomic sequences in human brainstem nuclei. npj Parkinson's Disease. 2023;9(1):25. doi: 10.1038/s41531-023-00467-3.

12. Farzad F, zahra M, Razieh F. Encephalitis Associated with COVID-19 in a7-Year-Old Boy: A Case Report. Case Reports in Clinical Practice. 2022;6(6):246-50. doi: 10.18502/crcp.v6i6.9171.

13. Moriguchi T, Harii N, Goto J, Harada D, Sugawara H, Takamino J, et al. A first case of meningitis/encephalitis associated with SARS-Coronavirus-2. International Journal of Infectious Diseases. 2020;94:55-8. doi: <https://doi.org/10.1016/j.ijid.2020.03.062>.

14. Elmakaty I, Ferih K, Karen O, Ouda A, Elsabagh A, Amarah A, et al. Clinical Implications of COVID-19 Presence in CSF: Systematic Review of Case Reports. Cells. 2022;11(20):3212. Epub 2022/10/28. doi: 10.3390/cells11203212. PubMed PMID: 36291083; PubMed Central PMCID: PMCPMC9600635.

15. Abdolahi S, Ashayeri Ahmadabad R, Gorji A, Mirzaasgari Z. Status epilepticus and the presence of SARS-COV-2 in the cerebrospinal fluid: A case report. Clin Case Rep. 2022;10(8):e6214. Epub 2022/08/13. doi: 10.1002/ccr3.6214. PubMed PMID: 35957773; PubMed Central PMCID: PMCPMC9361715.

16. Ayuningtyas T, Natadidjaja RI, Octaviani C, Sahli F, Adlani H. Confirmed severe acute respiratory syndrome coronavirus 2 encephalitis in cerebrospinal fluid: a case report. J Med Case Rep. 2022;16(1):154. Epub 2022/04/15. doi: 10.1186/s13256-022-03376-w. PubMed PMID: 35418102; PubMed Central PMCID: PMCPMC9007575.

17. Viszlayova D, Sojka M, Dobrodenkova S, Szabo S, Bilec O, Turzova M, et al. SARS-CoV-2 RNA in the Cerebrospinal Fluid of a Patient with Long COVID. Ther Adv Infect Dis. 2021;8:20499361211048572. Epub 2021/10/19. doi: 10.1177/20499361211048572. PubMed PMID: 34659752; PubMed Central PMCID: PMCPMC8511908.

18. Luis MB, Liguori NF, Lopez PA, Alonso R. SARS-CoV-2 RNA detection in cerebrospinal fluid: Presentation of two cases and review of literature. Brain Behav Immun Health. 2021;15:100282. Epub 2021/06/15. doi: 10.1016/j.bbih.2021.100282. PubMed PMID: 34124700; PubMed Central PMCID: PMCPMC8184365.

19. Dang TQ, La DT, Tran TN. Myeloencephalitis as the only presentation of Omicron SARS-CoV-2 infection. BMJ Case Rep. 2022;15(11):e251922. Epub 2022/11/16. doi: 10.1136/bcr-2022-251922. PubMed PMID: 36379626; PubMed Central PMCID: PMCPMC9668029.

20. Benameur K, Agarwal A, Auld SC, Butters MP, Webster AS, Ozturk T, et al. Encephalopathy and Encephalitis Associated with Cerebrospinal Fluid Cytokine Alterations and Coronavirus Disease, Atlanta, Georgia, USA, 2020. Emerg Infect Dis. 2020;26(9):2016-21. Epub 2020/06/04. doi: 10.3201/eid2609.202122. PubMed PMID: 32487282; PubMed Central PMCID: PMCPMC7454059.

21. Huang YH, Jiang D, Huang JT. SARS-CoV-2 Detected in Cerebrospinal Fluid by PCR in a Case of COVID-19 Encephalitis. Brain Behav Immun. 2020;87:149. Epub 2020/05/11. doi: 10.1016/j.bbi.2020.05.012. PubMed PMID: 32387508; PubMed Central PMCID: PMCPMC7202824.
